# Serum Amyloid A Induces a Vascular Smooth Muscle Cell Phenotype Switch through the p38 MAPK Signaling Pathway

**DOI:** 10.1155/2017/4941379

**Published:** 2017-05-31

**Authors:** Xincai Zhang, Jinqin Chen, Shixun Wang

**Affiliations:** ^1^Department of Cardiology II, Weifang People's Hospital, Kuiwen District, Weifang, Shandong 261041, China; ^2^Department of Sterilization and Supply, Weifang People's Hospital, Kuiwen District, Weifang, Shandong 261041, China

## Abstract

Atherosclerosis is an important pathological condition which is accompanied by a vascular smooth muscle cell (VSMC) phenotype switch toward a synthetic phenotype. As an acute-phase protein, Serum Amyloid A (SAA) is thought to have a close relationship to atherosclerosis development. However, no study has investigated the direct effect of SAA on the VSMC phenotype switch, as well as the underlying mechanisms. The purpose of our study was to explore the effect of SAA on the VSMC phenotype switch and the potential mechanisms involved. In our study, we found that SAA induced the VSMC phenotype switch which reduced expression of the smooth muscle cell (SMC) marker and enhanced expression of the matrix synthesis related marker. The proliferative ability of VSMCs was also increased by SAA treatment. Furthermore, our research found that SAA activated the ERK1/2 and p38 MAPK signaling pathways. Finally, by applying the ERK1/2 and p38 inhibitors, U0126 and SB203580, we demonstrated that the SAA-induced VSMC phenotype switch was p38-dependent. Taken together, these results indicated that SAA may play an important role in promoting the VSMC phenotype switch through the p38 MAPK signaling pathway.

## 1. Introduction

Cardiovascular disease (CVD) is the leading cause of morbidity and mortality in the developed world. Atherosclerosis is an important pathological condition in the development and progression of CVD [1, 2]. It is accompanied by abnormal growth of VSMCs and extracellular matrix synthesis [3]. VSMCs are highly plastic, existing in different phenotypic states, such as contractile and synthetic phenotypes [3]. Contractile VSMCs exhibit quiescence and the contractile phenotype expresses high levels of contractile proteins such as smooth muscle *α*-actin (*α*-SMA), h1-calponin, and SM22*α*. Synthetic VSMCs possess higher proliferative and extracellular matrix synthesis capacity and lose contractile ability [4]. Studies further demonstrated that VSMCs undergo transcriptionally regulated reversible differentiation in growth and injured blood vessels [5]. Therefore, in response to injury, the contractile VSMC phenotype switch (i.e., dedifferentiation) toward a synthetic phenotype is an important step which leads to vascular lesions [6]. So targeting the VSMC phenotype switch could be an important strategy in atherosclerosis therapy.

Atherosclerosis is a chronic inflammatory disease, and several inflammatory markers are useful in identifying the CVD risk. Among them is Serum Amyloid A (SAA), an acute-phase protein which is principally synthesized by the liver in response to acute inflammatory stimuli. SAA is also expressed in other tissues, such as synovial, arterial, and adipose tissue [7]. Previous studies found that SAA is an inflammatory marker which is positively and significantly associated with CVD [8, 9]. Accumulating evidence demonstrated the participation of SAA in atherogenesis. SAA can promote monocyte and polymorphonuclear leukocyte migration and adhesion [10]. It also increases high-density lipoprotein (HDL) binding to macrophages and endothelial cells [11]. Another study showed that SAA induces endothelial dysfunction [12]. Previous reports also found that it can reduce VSMC lipid biosynthesis [13] and activate VSMC IL-1*β* expression [14]. These findings implied the close association between SAA and atherosclerosis development. However, to the best of our knowledge, no study has investigated the relationship between SAA and the VSMC phenotype switch, as well as the underlying mechanisms.

MAPKs and Akt pathways play a crucial role in modulating the VSMC phenotype switch [15–17]. SAA was demonstrated to activate the Akt pathways and three MAPKs, the extracellular signal-regulated kinase 1/2 (ERK1/2), p38, and c-Jun N-terminal kinase (JNK) [18, 19]. Thus, our study examined the effect of SAA on the VSMC phenotype switch and explored the mechanisms involved.

## 2. Materials and Methods

### 2.1. Animals and Cell Culture

Male Sprague Dawley (SD) rats (8 weeks old) were purchased from Shanghai Slac Laboratory Animal Co. Ltd. Before tissue harvesting the rats were euthanized by an overdose of sodium pentobarbital (100 mg/kg) by intraperitoneal (IP) injection. All animal experiments were carried out according to the National Institutes of Health Guide for the Care and Use of Laboratory Animals and were approved by the Shandong University of Laboratory Animals Care and Use Committee.

Rat aortic smooth muscle cells (RASMCs) were isolated and cultured as previously described [20]. Briefly, the thoracic aortas of SD rats were removed and washed in PBS and incubated in Dulbecco's modified Eagle's medium (DMEM) along with 300 unit/mL of collagenase type II (Worthington, USA) for 30 min. Then, the surrounding connective tissues and adventitia were dissected away, and the endothelium was removed by scraping off the cell layer with sterile scalpel blades. The dissected tunica media tissues were incubated with 300 unit/ml collagenase type II in DMEM for 2-3 h until all tissue was digested. Cells were cultured in DMEM supplemented with 10% fetal bovine serum. Recombinant SAA protein (a consensus molecule of the SAA1 and SAA2, endotoxin level less than 0.1 ng/*μ*g) was purchased from PeproTech (USA). U0126 and SB203580, selective inhibitors of ERK and p38, respectively, were purchased from Sigma-Aldrich (USA).

### 2.2. RNA Extraction and Quantitative Reverse Transcription-PCR

Total RNA was extracted from RASMCs by using the TRIzol reagent (Invitrogen, USA) and then reverse-transcribed to cDNA with the RevertAid First Strand cDNA Synthesis kit (Thermo scientific) according to the manufacturer's instructions. qPCR analysis was performed by using a LightCycler (Bio-Rad, USA). Melting curves were assessed to confirm the specificity of the products generated for each set of primers. Expression levels of target genes were normalized by concurrent measurement of GAPDH mRNA levels. All primers used are listed in Table 1.

### 2.3. Western Blot

Total cell protein concentrations were determined using the BCA protein assay kit (Pierce, Rockford, IL, USA). Equal amounts of protein from cell lysates were loaded in 12% SDS-PAGE gels. After electrophoresis, proteins were transferred to PVDF membranes (Millipore, USA), blocked with 5% fat-free milk at room temperature for 1 h, and incubated with the indicated primary antibodies overnight at 4°C. Then, the membranes were incubated with HRP-conjugated secondary antibodies for 1 h at room temperature. Immune complexes were detected with ECL reagents, and the blots were quantified by densitometric analysis using the Alpha Imager 2200 (USA).

The primary antibodies were anti-ERK1/2 (cat: 4695, Cell Signaling Technology, USA), anti-phosphor-ERK1/2 (cat: 4370, Cell Signaling Technology, USA), anti-p38 (cat: 8690, Cell Signaling Technology, USA), anti-phosphor-p38 (cat: 4631, Cell Signaling Technology, USA), anti-JNK (cat: 9252, Cell Signaling Technology, USA), anti-phosphor-JNK (cat: 4668, Cell Signaling Technology, USA), anti-Akt (cat: 4685, Cell Signaling Technology, USA), anti-phosphor-Akt (cat: 4060, Cell Signaling Technology, USA), anti-MMP2 (cat: 13132, Cell Signaling Technology, USA), anti-MMP9 antibodies (cat: 13667, Cell Signaling Technology, USA), anti-Collagen I (cat: ab34710, Abcam, USA), anti-h1-calponin (cat: ab46794, Abcam, USA), anti-SM22*α* (cat: ab10135, Abcam, USA), anti-*α*-SMA (cat: A5228, Sigma-Aldrich, USA), anti-GAPDH (cat: G9545, Sigma-Aldrich, USA), and anti-CRBP-1 (cat: sc-271208, Santa Cruz, USA).

### 2.4. Immunofluorescent Staining

After incubation with SAA, the RASMCs were fixed with 4% paraformaldehyde, permeabilized with 0.5% Triton X-100, and incubated with 5% normal goat serum for 1 h. Then, the cells were incubated with rabbit anti-h1-calponin, anti-*α*-SMA, or anti-SM22*α* overnight at 4°C. This was followed by incubation with Alexa-Fluor-488-conjugated goat anti-rabbit IgG or Alexa-Fluor-594-conjugated goat anti-rabbit IgG for 1 h at room temperature. The cells were further incubated with 4′,6-diamidino-2-phenylindole (DAPI)/PBS (1 : 5000, Sigma) for 3 min at room temperature. Finally, images were acquired by using a Nikon Eclipse 80i fluorescent microscope.

### 2.5. [3H] Thymidine Uptake

RASMCs grown in DMEM to a 50% confluent state were incubated with or without SAA (10 *μ*g/ml) for 3 days and then cultured in serum-free DMEM for 24 h. The cells were treated with or without 10% FBS for 40 h with [3H]thymidine (1 *μ*Ci/ml) being added during the last 24 h of culture. DNA synthesis was measured by [3H] thymidine uptake and normalized by the number of cells in each group.

### 2.6. Scratch Wound Healing Assay

A scratch wound healing assay was used to assess the migration ability of the RASMCs. In brief, RASMCs were incubated with or without SAA (10 *μ*g/ml) for 3 days. When confluency was reached, a straight scratch with a 200 *µ*l pipette tip was made in each well. After 24 h of incubation, the wound healing areas were photographed and then the distance between the two cell edges was analyzed by ImageJ software.

### 2.7. Statistics

The data are expressed as the mean ± standard deviation (SD). All of the experiments were repeated three times. Student's *t*-test was used for comparing differences of different groups. Comparisons among values of multiple groups were performed by one-way analysis of variance (ANOVA). Differences were considered to be significant at *P* < 0.05.

## 3. Results

### 3.1. SAA Induces RASMC Dedifferentiation

**Figure 1:**
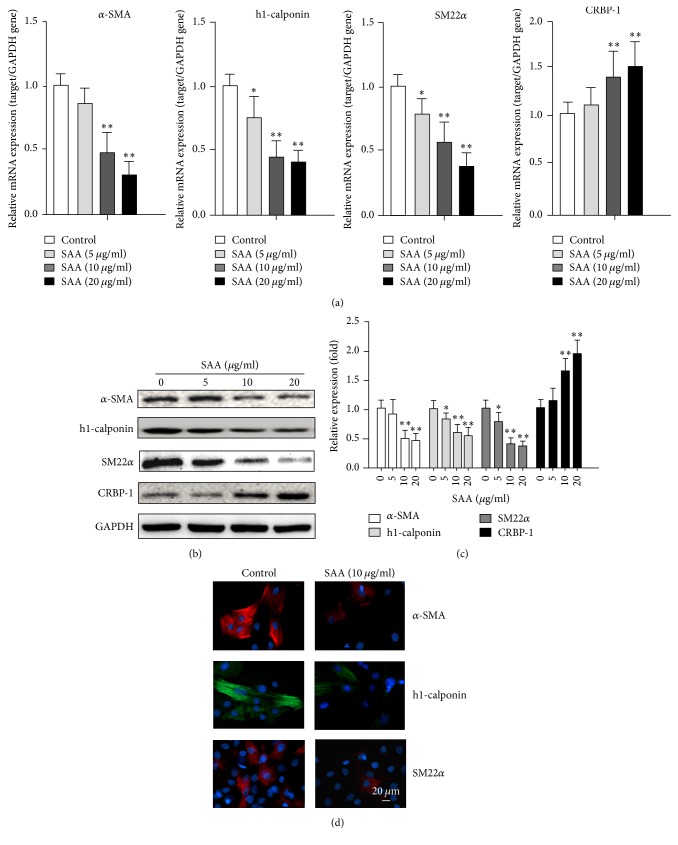
SAA induces RASMC dedifferentiation. The RASMCs were treated with SAA at indicated concentrations for 72 h. (a) mRNA expression of the smooth muscle cell (SMC) related gene (*α*-SMA, h1-calponin, and SM22*α*) and CRBP-1 was examined by qPCR analysis. (b) Expression of the smooth muscle cell (SMC) related markers (*α*-SMA, h1-calponin, and SM22*α*) and CRBP-1 was detected by Western blot analysis. (c) Statistical analysis of the Western blot results. (d) Expression of *α*-SMA, h1-calponin, and SM22*α* was detected by immunofluorescent staining. ^*∗*^*P* < 0.05, ^*∗∗*^*P* < 0.01 versus the control group. Data shown are means ± SD from three independent experiments.

To explore the role of SAA on the RASMC phenotype switch, RASMCs were treated with SAA (0, 5, 10, and 20 *μ*g/ml) for 72 h. As shown in Figure 1(a), SAA suppressed the smooth muscle cell (SMC) gene (*α*-SMA, h1-calponin, and SM22*α*) and induced SMC dedifferentiation marker cellular retinol binding protein 1 (CRBP-1) mRNA expression in a dose-dependent manner. In addition, Western blot was used to evaluate the protein expression of SMC markers (*α*-SMA, h1-calponin, SM22*α*, and CRBP-1). As shown in Figures 1(b) and 1(c), in accordance with the mRNA level, the protein level of SMC markers (*α*-SMA, h1-calponin, and SM22*α*) was also significantly downregulated when treated with SAA, while the protein level of CRBP-1 was upregulated. Immunofluorescent staining was also used to detect expression of SMC markers. After SAA (10 *μ*g/ml) treatment for 24 h, RASMCs were stained with *α*-SMA, h1-calponin, and SM22*α* and analyzed by fluorescent microscopy. As shown in Figure 1(d), 10 *μ*g/ml SAA could effectively reduce expression of *α*-SMA, h1-calponin, and SM22*α*. These data indicated that SAA could induce RASMC dedifferentiation.

### 3.2. SAA Promotes RASMCs toward a Synthetic Phenotype

**Figure 2:**
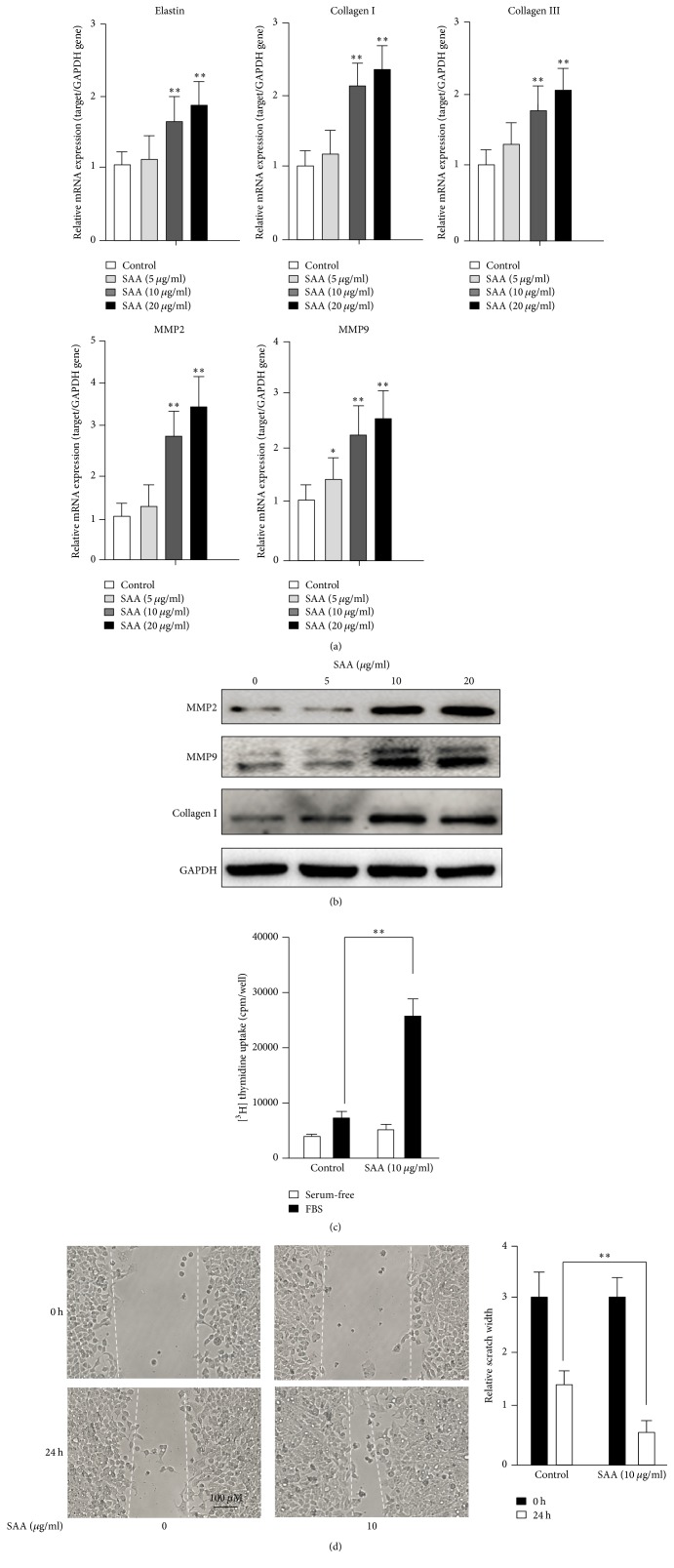
SAA promotes RASMCs toward a synthetic phenotype. The RASMCs were treated with SAA at indicated concentrations for 72 h. (a) mRNA expression of the matrix synthesis related gene was examined by qPCR analysis. (b) Expression of matrix synthesis related markers was detected by Western blot analysis. (c) The proliferative capacity was accessed by [3H] thymidine uptake. (d) The migratory capacity was accessed by the migration assay. ^*∗*^*P* < 0.05, ^*∗∗*^*P* < 0.01 versus the control group. Data shown are means ± SD from three independent experiments.

To further explore the role of SAA on the RASMC phenotype switch, RASMCs were treated with SAA (0, 5, 10, and 20 *μ*g/ml) for 72 h and the mRNA and protein levels of the matrix synthesis related markers were detected by qPCR and Western blot. As shown in Figure 2(a), when treated with SAA, mRNA expression of the matrix synthesis related markers (Elastin, Collagen I, Collagen III, MMP2, and MMP9) was increased in a dose-dependent manner. Also, the protein expression of the matrix synthesis related markers (MMP2, MMP9, and Collagen I) was upregulated when treated with SAA in a dose-dependent manner (Figure 2(b)). Furthermore, [3H] thymidine uptake and migration assay were used to evaluate the effect of SAA on the RASMC phenotype switch. As shown in Figures 2(c) and 2(d), SAA-treated RASMCs exhibited a more proliferative and migratory phenotype. The proliferative and migratory property was positively associated with matrix synthesis ability. These results further implied that SAA promoted RASMC dedifferentiation into a synthetic phenotype.

### 3.3. SAA Promotes p38 and ERK1/2 Phosphorylation in RASMCs

**Figure 3:**
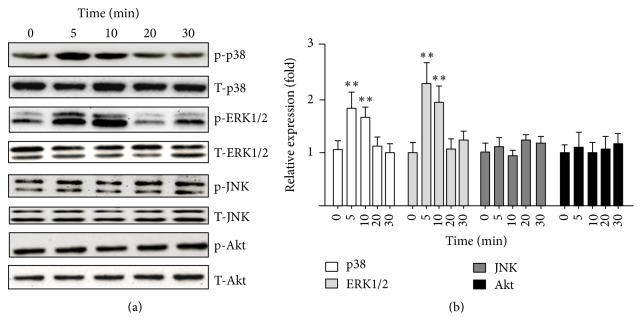
SAA promotes p38 and ERK1/2 phosphorylation in RASMCs. RASMCs were treated with 10 *µ*g/ml SAA at indicated time points. (a) Phosphorylated and total p38, ERK1/2, JNK, and Akt levels in cell lysates were detected by Western blot. (b) Statistical analysis of the Western blot results. ^*∗∗*^*P* < 0.01 versus the control group. Data shown are means ± SD from three independent experiments.

Previous studies showed the pivotal role of MAPK and Akt signaling pathways in the VSMC phenotype switch [15–17]. To explore the potential mechanism of the SAA-induced RASMC phenotype switch, the ERK1/2, p38, JNK, and Akt signaling pathways were detected. As shown in Figure 3(a), ERK1/2 and p38 phosphorylation were activated when treated with 10 *μ*g/ml SAA. The activation was statistically significant, as quantified by densitometry (Figure 3(b)), whereas the JNK and Akt phosphorylation were not affected by SAA treatment. Therefore, these results indicated that ERK1/2 and p38 signaling pathways may be involved in the SAA-induced RASMC phenotype switch.

### 3.4. The ERK1/2 Signaling Pathway Is Not Involved in the SAA-Induced Phenotype Switch in RASMCs

**Figure 4:**
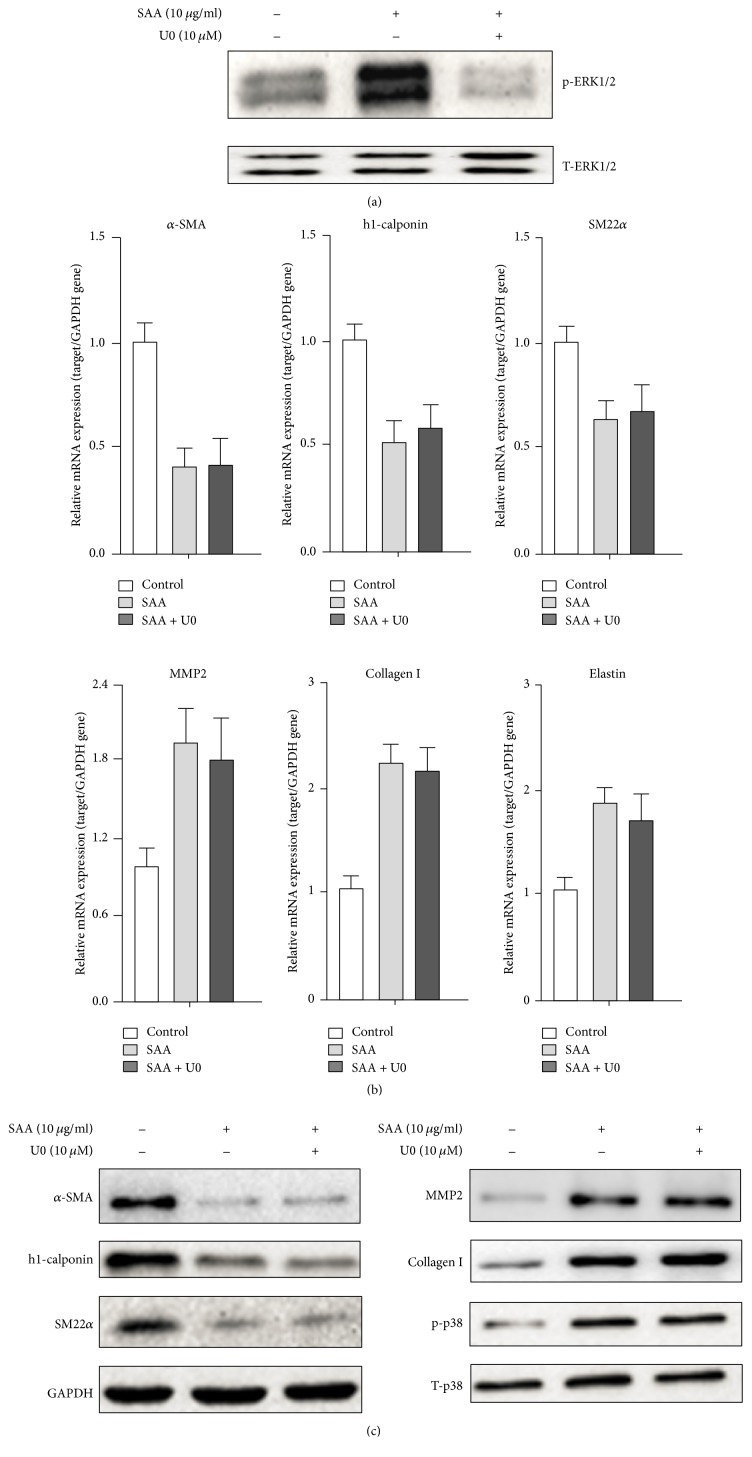
Inhibition of ERK1/2 does not affect the SAA mediated RASMC phenotype switch. RASMCs were pretreated with the ERK1/2 inhibitor U0126 (10 *μ*M) for 30 min followed by SAA treatment. (a) Western blot analysis of phosphorylated and total ERK1/2 protein expression. (b) qPCR analysis of the effect of the ERK1/2 inhibitor on SAA-induced RASMC phenotype modulation. (c) Western blot analysis of the effect of ERK1/2 inhibitor on SAA-induced RASMC phenotype modulation and the phosphorylated and total p38. Data shown are means ± SD from three independent experiments.

To further explore whether ERK1/2 is involved in the SAA-induced phenotype switch, U0126 (a ERK1/2 inhibitor) was introduced to block ERK1/2 phosphorylation (Figure 4(a)). As shown in Figure 4(b), at the mRNA level, inhibition of ERK1/2 phosphorylation with U0126 did not affect the SAA-induced downregulation of the SMC related gene (*α*-SMA, h1-calponin, and SM22*α*) and upregulation of the matrix synthesis related gene (MMP2, Collagen I, and Elastin). As shown in Figure 4(c), similar to the mRNA level, inhibition of ERK1/2 phosphorylation also did not affect the SAA-induced downregulation of SMC related markers (*α*-SMA, h1-calponin, and SM22*α*) and upregulation of matrix synthesis markers (MMP2 and Collagen I) at the protein level. Also, the phosphorylation of p38 was not affected by U0126 (Figure 4(c)). These results indicated that the SAA-induced RASMC phenotype switch might be ERK1/2-independent.

### 3.5. The p38 Signaling Pathway Is Involved in the SAA-Induced Phenotype Switch in RASMCs

**Figure 5:**
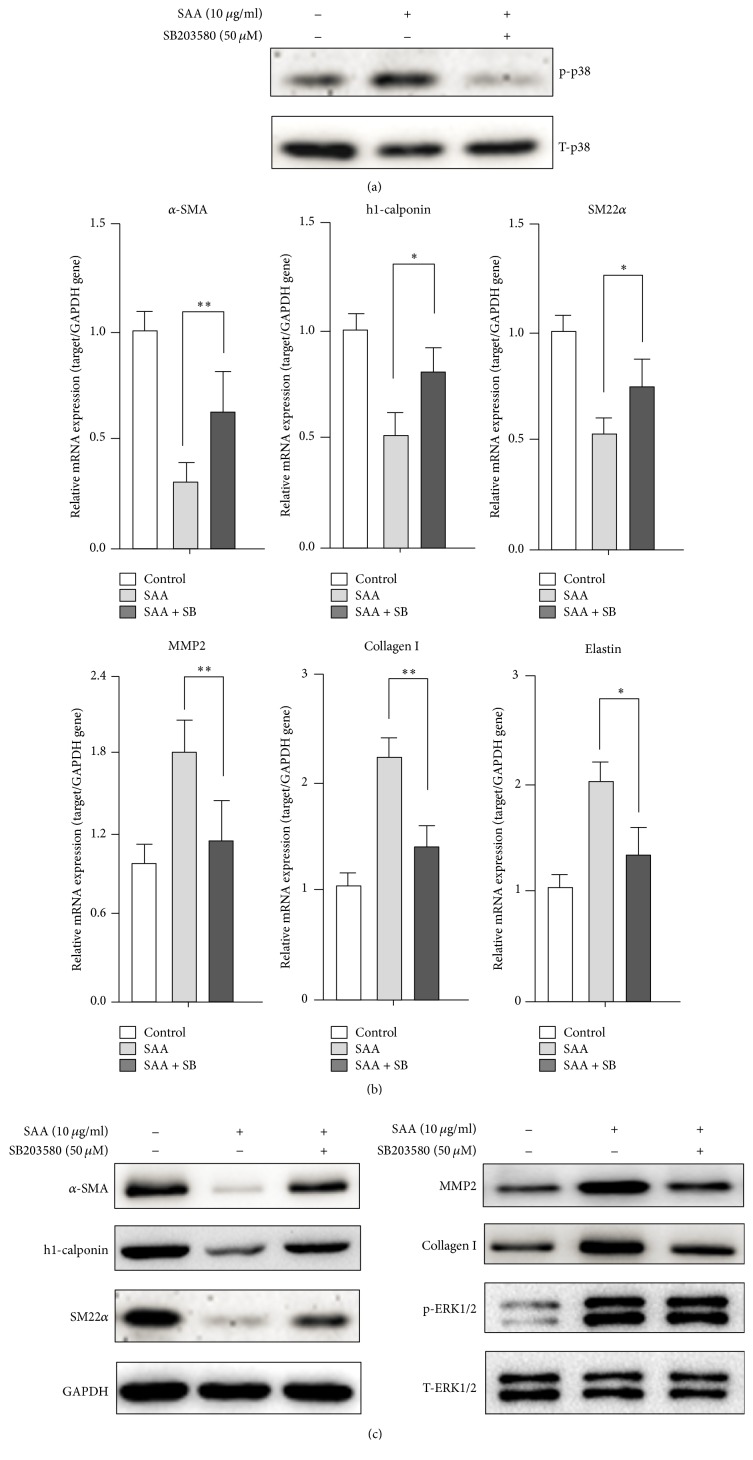
Inhibition of p38 reverses the SAA mediated RASMC phenotype switch. RASMCs were pretreated with the p38 inhibitor SB203580 (50 *μ*M) for 30 min followed by SAA treatment. (a) Western blots analysis of phosphorylated and total p38 protein expression. (b) qPCR analysis of the effect of the p38 inhibitor on SAA-induced RASMC phenotype modulation. (c) Western blot analysis of the effect of the p38 inhibitor on SAA-induced RASMC phenotype modulation and the phosphorylated and total ERK1/2. ^*∗*^*P* < 0.05, ^*∗∗*^*P* < 0.01 versus SAA-treated group. Data shown are means ± SD from three independent experiments.

To further explore whether p38 is involved in the SAA-induced phenotype switch, SB203580 (a p38 inhibitor) was applied to block p38 phosphorylation (Figure 5(a)). As shown in Figure 5(b), at the mRNA level, inhibition of p38 phosphorylation with SB203580 reversed the SAA-induced downregulation of the SMC related gene (*α*-SMA, h1-calponin, and SM22*α*) and upregulation of the matrix synthesis related gene (MMP2, Collagen I, and Elastin). As shown in Figure 5(c), similar to the mRNA level, inhibition of p38 phosphorylation also reversed the SAA-induced downregulation of SMC related markers (*α*-SMA, h1-calponin, and SM22*α*) and upregulation of matrix synthesis markers (MMP2 and Collagen I) at the protein level. In addition, the phosphorylation of ERK1/2 was not affected by SB203580 (Figure 5(c)). Taken together, these results implied that the SAA-induced RASMC phenotype switch might be p38-dependent. These results indicated that blocking p38 activation could significantly reverse the SAA-induced RASMC phenotype switch.

## 4. Discussion

In the present study, we reported several novel findings about SAA. SAA induced the VSMC phenotype switch toward a synthetic phenotype and activated ERK1/2 and p38 MAPK pathways in VSMCs. In addition, we demonstrated that SAA induced the VSMC phenotype switch through the p38 MAPK signaling pathway.

In the acute-phase response, SAA is mainly produced by the liver, leading to increased secretion. Then SAA is catabolized by the liver to a lower level. However, in chronic inflammatory diseases, such as atherosclerosis, production of SAA can be persistently increased [7]. Evidence also showed that other tissues, such as synovial, arterial, and adipose tissue, have the same ability [7]. Only a low level of SAA is present in the serum of healthy individuals [21], while a remarkable elevation of SAA can be found in the serum of CVD patients [8, 9]. Atherosclerosis is an inflammatory disease which is characterized by dynamic interactions between smooth muscle cells, endothelial cells, and macrophages [22]. Previous studies found that SAA can promote atherosclerosis development by recruiting monocytes and polymorphonuclear leukocytes [10], increasing HDL binding to macrophages and endothelial cells [11], inducing endothelial dysfunction [12], and lessening VSMC lipid biosynthesis [13]. Furthermore, Belmokhtar et al. demonstrated that SAA could modulate the functions of VSMCs in uremia-related atherogenesis [23]. To the best of our knowledge, the present study is the first to focus on the relationship between SAA and the VSMC phenotype switch. To determine the effect of SAA on the VSMC phenotype switch, RASMCs were cultured and stimulated by SAA. We found that SAA could significantly induce the VSMC phenotype switch toward a proliferative, synthetic phenotype.

It was demonstrated that the VSMC phenotype switch toward a proliferative, synthetic phenotype participated in atherosclerosis [24]. Previous studies found the role of the MAPKs pathway in SAA-induced various cell functions, such as mononuclear cell CCL20 secretion [25], endothelial cell function and angiogenesis [26], and the macrophage regulated inflammatory response [27]. In the present study, we evaluated the MAPKs (ERK1/2, p38, and JNK) and Akt expression in VSMCs. We found that SAA treatment activated the phosphorylation of ERK1/2 and P38 MAPK, while phosphor-JNK and phosphor-Akt were not affected. To further explore the mechanism involved in SAA-induced phenotype switch in VSMCs, we blocked ERK1/2 and P38 activity by their inhibitors. We found that blockade of p38 MAPK significantly reversed the SAA-induced VSMC phenotype switch. These results further indicated that p38 MAPK is pivotal for the VSMC phenotype switch. However, blockade of ERK1/2 did not reverse the SAA-induced VSMC phenotype switch. These results implied that the SAA-induced VSMC phenotype switch is ERK1/2-independent.

In conclusion, the present study for the first time provides evidence that SAA induces the VSMC phenotype switch. Furthermore, the effect of SAA in the VSMC phenotype switch is modulated through the p38 MARK pathway. Thus, our findings indicated that inhibiting SAA is a potential way to restrain the progression of cardiovascular disease.

## Figures and Tables

**Table 1:** Primers for qPCR.

Primers	Gene access #	Forward; 5′—3′	Reverse: 5′—3′	Product length (bp)
*α*SMA	NM_031004.2	CGCCATCAGGAACCTCGAGA	CAAAGCCCGCCTTACAGA	103
h1-Calponin	NM_031747.1	ACATCATTGGCCTACAGATG	CAAAGATCTGCCGCTTGGTG	204
SM22*α*	M83107.1	CCACAAACGACCAAGCCTTTT	CGGCTCATGCCATAGGATG	86
CRBP-1	XM_016044.2	CAGATGAAGCTACTTGTATGGGCTTC	GGCAAAGCGAAGCTTTGGCATC	253
Elastin	NM_012722.1	CATCGGTGGCTTAGGAGTCT	GAAGACCGACACCAGGAACT	101
MMP2	NM_031054.2	GACCTTGACCAGAACACCATCG	GCTGTATTCCCGACCGTTGAAC	270
MMP9	NM_031055.1	AAGGATGGTCTACTGGCAC	AGAGATTCTCACTGGGGC	280
Collagen I	NM_053304.1	GGCGAGTGCTGTCCTTTCTG	CTTCCCCATCATCTCCGTTCTCTTCCCCAT	434
Collagen III	AJ005395.1	AGATCATGTCTTCACTCAAGTC	TTTACATTGCCATTGGCCTAG	465
GAPDH	NM_017593963.1	AGGTCGGTGTGAACGGATTTG	GGGGTCGTTGATGGCAACA	95
